# DR6 Augments Colorectal Cancer Cell Growth, Invasion, and Stemness by Activating AKT/NF-κB Pathway

**DOI:** 10.1007/s10528-024-10673-0

**Published:** 2024-03-13

**Authors:** Jing Jia, Yisen Huang, Qiwei Chen, Jianbin Hou, Yan Liu, Lifeng Xie, Xinyu Li, Chunkang Yang

**Affiliations:** 1 Department of Gastrointestinal Surgery, Quanzhou First Hospital Affiliated to Fujian Medical University, 250 Dongjie, Licheng District, Quanzhou, 362002 Fujian China; 2 Department of Gastroenterology, Quanzhou First Hospital Affiliated to Fujian Medical University, Quanzhou, 362002 Fujian China; 3 Department of Gastrointestinal Surgery, Fujian Cancer Hospital, Clinical Oncology School of Fujian Medical University, Fuma Road Fengban, Jin’an District, Fuzhou, 362002 Fujian China

**Keywords:** Death receptor 6, Colorectal cancer, Proliferation, Migration, Invasion, Stemness features

## Abstract

This study aims to elucidate the role and mechanisms of Death Receptor 6 (DR6), a member of the tumor necrosis factor receptor superfamily, in the malignant progression of colorectal cancer (CRC). The association of DR6 expression levels and CRC patient survival was examined using the CRC cohort data from GEPIA database. The functional role of DR6 in CRC cells was investigated by performing loss-of-function and gain-of-function experiments based on CCK-8 proliferation assay, transwell migration and invasion assay, and sphere-forming assays. Xenograft model of CRC cells in nude mouse was established to evaluate the impact of DR6 knockdown on CRC tumorigenesis. Elevated expression of DR6 was correlated with an unfavorable prognosis in CRC patients. In vitro functional assays demonstrated that silencing DR6 considerably suppressed the proliferation, migration, invasion, and stemness of CRC cells, whereas its overexpression showed an opposite effect. DR6 knockdown also attenuated tumor formation of CRC cells in the nude mice. Mechanistically, silencing DR6 reduced the phosphorylation of AKT and NF-κB in CRC cells, and the treatment with an AKT activator (SC79) abrogated the inhibitory effects of DR6 knockdown on the malignant features of CRC cells. Our data suggest that DR6 contributes to the malignant progression of CRC by activating AKT/NF-κB pathway, indicating its clinical potential as a prognostic marker and therapeutic target for CRC.

## Introduction

Colorectal cancer (CRC) is the fourth most common malignancy and one of the leading causes of cancer-related deaths worldwide, with a gradually increasing incidence and high mortality rate (Wu et al. 2020; Li et al. 2021; Islam et al. 2022; Sun et al. 2021). The 5-year and 10-year survival rates of CRC are 65% and 58%, respectively, which poses a serious threat for human health (Wu et al. 2020; Li et al. 2021; Islam et al. 2022; Sun et al. 2021). Currently, surgical resection combined with chemotherapy and radiotherapy is the mainstream option for CRC treatment, which can significantly improve the survival of CRC patients (Wu et al. 2020; Li et al. 2021; Islam et al. 2022; Sun et al. 2021). However, the prognosis in patients diagnosed with advanced CRC remains poor, and the postoperative cancer recurrence and metastasis are the leading causes of mortality (Ma et al. 2022; Yang et al. 2020). With the rapid development and widespread application of second-generation sequencing technology, the molecular profiles of many cancers, including CRC, have been comprehensively characterized. This has enabled the application of molecular biomarkers as predictive and prognostic tools to assess the clinical outcome of CRC patients (Zhu et al. 2021; Martini et al. 2020). Li et al. reported that the downregulation of MEIS1 expression in CRC was associated with a poor prognosis. MEIS1 functions as a tumor suppressor to inhibit CRC cell growth and tumor formation by suppressing DNA damage repair and increasing the sensitivity to oxaliplatin (Li et al. 2022). Further, Long et al. conducted a review on the dysregulation of circular RNAs (circRNAs), their functions, and clinical significance in CRC progression (Long et al. 2021). A growing body of evidence indicates that circRNAs have the potential to serve as biomarkers and therapeutic targets for the diagnosis and prognostic prediction of CRC (Long et al. 2021). These findings highlight the potential of novel molecular targets for clinical management of CRC.

Death receptor 6 (DR6) is a member of the tumor necrosis factor receptor superfamily (TNFRSF), which plays a crucial role in regulating cell growth, differentiation, apoptosis, and survival (Ren et al. 2022). However, the aberrant activation of DR6 has been widely reported in the development and progression of different pathophysiological conditions, including rheumatoid arthritis, inflammatory bowel disease, Alzheimer's disease, type 2 diabetes, and cancers (Ren et al. 2022). For example, DR6 has been characterized as a key regulator of axonal degeneration and a therapeutic target for prion-associated neurodegenerative diseases (Wang et al. 2015). A study by Wu et al. reported that DR6 is involved in regulating the proliferation and invasion of head and neck squamous cell carcinoma by acting as a downstream target of miR-20a-5p (Wu et al. 2018). DR6 was also found to be upregulated in pancreatic tumor tissues and cell lines, and low expression of DR6 was associated with a significant survival advantage and correlated with a low level of immune infiltration (Xu et al. 2022). DR6 has also been recognized as an oncogene which is highly expressed in glioma, ovarian cancer, sarcoma, and melanoma (Stegmann et al. 2022; Shi et al. 2018; Yang et al. 2012, 2016). However, the potential role of DR6 in dictating the malignancy and progression of CRC remains to be investigated.

In this study, we first adopted the Gene Expression Profiling Interactive Analysis (GEPIA) database to analyze the expression levels of DR6 in CRC tumor samples and normal tissues. The expression pattern of DR6 in CRC tumors was verified by qRT-PCR and Western blotting in clinical samples. The functional role of DR6 on the malignant phenotype of CRC cells were investigated by silencing or overexpressing DR6. In addition, we also explored the involvement of AKT/NF-κB pathway in the effect of DR6.

## Materials and Methods

### Patients and Samples

Tumor tissues and normal para-cancerous tissues were collected from 100 patients diagnosed with CRC by surgery. Clinical data, including patient age, gender, histological stage of tumor samples, pathologic diagnosis, and metastasis, were collected. None of the patients received radiotherapy or chemotherapy prior to surgery. The collected specimens were snap-frozen in liquid nitrogen and stored at -80 °C. The usage of human samples had been approved by the Ethics Committee of Clinical Oncology School of Fujian Medical University, Fujian Cancer Hospital. Informed consent was obtained from each enrolled patient.

### qRT-PCR

Total cellular RNA was extracted using Trizol reagent (Ambion, 15,596,026, Carlsbad, CA, USA). cDNA was synthesized by reverse transcription, and PCR amplification was conducted using SYBR FAST qPCR Master Mix (KAPA Biosystems, KM4101, MA, USA). The following cycling conditions were used in qPCR: 95 °C for 3 min, 40 cycles of 95 °C for 5 s, 56 °C for 10 s, and 72 °C for 25 s. The following primer sequences were used in qRT-PCR analysis: *DR6* forward primer: 5'-ATCCGGAAAAGCTCGAGGAC-3', and reverse primer: 5'-CTTCCCACTTGGGCTGCTAC-3'. *GAPDH* forward primer: 5'-CGGATTGGTCGTATTGGGG-3', and reverse primer: 5'-CTGGAAGATGGTGATGGGATT-3'. Ct values were obtained at the end of the reaction and relative gene expression levels were determined using the 2^−∆∆Ct^ method with GAPDH as an internal reference.

### Cell Culture and Treatment

CRC cell lines (HCT116, LOVO, SW480, and SW620) and normal colonic epithelial cells FHC were obtained by the Shanghai Institute of Cell Research, Chinese Academy of Sciences (Shanghai, China). The cells were cultured in DMEM medium (Gibco, C11995500BT, CA, USA) supplemented with 10% fetal bovine serum (FBS) at 37 °C in a 5% CO_2_ incubator. AKT activator (SC79) (MedChemExpress, HY-18749, Shanghai, China) was used at 10 μM for intervention.

Cells with stable DR6 knockdown were generated using the lentiviral system, following the methodology described in a previous study (Zhong et al. 2020). The shRNA sequences targeting DR6, sh-DR6-#1 (5'-CCTATGTCTCTGAGCATTGTA-3'), sh-DR6-#2 (5'-CCTATGTCTCTGAGCATTGTA-3'), and sh-DR6-#3 (5'-CCGGGAGAAATGGATCTACTA-3'), along with the negative control sequence sh-NC (5'-GTATATCAGTGCTATTCGCCT-3'), were designed and synthetically generated by Genechem Co., Ltd. (Shanghai, China). These sequences were subsequently cloned into the pLenti-shRNA-GFP-puro lentiviral vector for further experimental applications. To produce lentiviral particles, 293 T cells (Cell Bank of the Chinese Academy of Sciences, Shanghai, China) were inoculated into a 10-cm culture dishes and allowed to grow until 60%-70% confluence. The lentiviral vector and packaging mixtures were delivered into 293 T cells using Lipofectamine 3000 (Invitrogen, 11,668–027, CA, USA). 48 h after transfection, viral particles were collected by centrifuging the cell culture supernatant at 2000 rpm for 15 min. To generate stable shRNA-mediated knockdown of DR6, 1 × 10^5^ cells were seeded in a 24-well plate to reach 50 ~ 60% confluence. The cells were transduced with lentiviral particles at a MOI (multiplicity of infection) = 5, in the presence of 10 µg polybrene (Sigma, tr-1003-g, Shanghai, China) for 48 h. Infected cells were further selected with 1.0 μg/mL puromycin for 14 days to eliminate the uninfected cells. qRT-PCR and Western blot were used to confirm the efficiency of shRNA-mediated knockdown.

To overexpress DR6, the transfection of DR6 expression vector was performed according to a previous study (Tian et al. 2020). The empty pcDNA3.1 plasmid and pcDNA3.1-RD6 expression plasmid were produced by Genechem (Shanghai, China). CRC cells in the logarithmic growth phase were inoculated in 6-well plates at 5 × 10^5^ cells/well for 24 h. Afterward, 4 µg of plasmid DNA was diluted with 250 µl of Opti-MEM (Gibco, 10,270–106, CA, USA) and mixed gently by aspiration. Then 10 µL of Lipofectamine 3000 (Invitrogen, 11,668–027, CA, USA) was diluted with 250 µl of Opti-MEM. The two solutions were combined and incubated for 15 min after ambient temperature before adding to the CRC cells. The cells were further cultivated in a 5% CO_2_ incubator at 37 °C for 48 h before subsequent experiments.

### CCK-8 Assay

Cells with/without DR6 knockdown or overexpression were inoculated into 96-well plates at a density of 5 × 10^3^ cells per well and incubated for 24 h. AKT activator (SC79) was applied at 10 μM. Cells were cultivated for 24, 48, 72, and 96 h, and 10 μl of CCK-8 solution (Solarbio, CA1210, Beijing, China) was added to each well at indicated time point for another 4-h incubation at 37 °C. Absorbance was measured at 450 nm using a microplate reader (Multiskan FC, Shanghai, China).

### Transwell Assays

For the cell migration assay, cells were adjusted to 1 × 10^5^ cells/ml and inoculated into the upper transwell chamber at 0.5 ml. The lower compartment was filled with 0.75 ml of culture medium containing 10% FBS. The plate was incubated at 37 °C for 48 h, and the migrating cells on the transmembrane were fixed by 4% formaldehyde solution (Macklin, F111934, Shanghai, China) for 20 min, followed by the staining with 0.25% crystal violet solution (Leagene Biotechnology, DA0061, Beijing, China) for 30 min. For the cell invasion assay, 80 µl of Matrigel gel (Shanghai Fusheng Industrial Co., Ltd., FS-79064, Shanghai, China) was spread in the upper chamber for coating at 37 °C for 30 min. The remaining experimental procedures were identical to the cell migration assay.

### Tumor Cell Spheroid Formation

The cell density was adjusted to 4 × 10^3^ cells/ml, and 200 μl of the cell suspension was inoculated into 24-well low attachment cell culture plates (WHB Scientific, 179,630, Shanghai, China). The plates were incubated at 37 °C in a 5% CO_2_ incubator for 7 days, and the medium was changed every two days. The formation of cell spheroids was observed and counted under an inverted microscope (BX5, Olympus, Tokyo, Japan).

### Western Blotting

Total proteins were extracted using radioimmunoprecipitation assay (RIPA) lysis buffer (Solarbio, R0030, Beijing, China) and the protein concentration was quantified using a bicinchoninic acid (BCA) assay kit (Solarbio, PC0020, Beijing, China). A total of 20 μg protein was separated using 12% polyacrylamide gel and separated protein samples were transferred to polyvinylidene fluoride membranes (Millipore, IPVH00010, MA, USA). The membranes were blocked using 5% non-fat milk at ambient temperature for 1 h and incubated with primary antibodies against DR6 (Sigma-Aldrich, SAB1407346, MO, USA), p-AKT (CST, 4060, MA, USA), AKT (Abcam, ab38449, Cambridge, UK), p-NF-κB (CST, 3033, MA, USA), NF-κB ( ABclonal, 4790, Wuhan, China), and GAPDH (CST, 5174, MA, USA) at 4 °C for 12 h. After washing, the membranes were further labeled with goat anti-rabbit IgG secondary antibody (Solarbio, K1034G-AF594, Beijing, China) for 1 h at ambient temperature. GAPDH was used as the loading control. An enhanced chemiluminescence (ECL) kit (Beyotime, P0018M, Beijing, China) was used to visualize protein banes on the membrane. The grayscale values of each group of protein bands were determined using Image J software (NIH, Bethesda, MA, USA).

### Xenograft Mouse Model

Balb/C Nude mice (female, 8-weeks old) were purchased from Hubei Experimental Animal Research Center (Hubei, China). The nude mice were randomly divided into 2 groups: the sh-NC group (mice injected with HCT116 cells carrying control shRNA) and the sh-DR6 group (mice injected with HCT116 cells carrying DR6 shRNA), with five mice in each group. A total number of 1 × 10^6^ HCT116 cells were injected subcutaneously into the left and right hind limbs. Tumor growth was observed every four days, and the long and short diameters of the tumors were recorded to calculate tumor volume. Tumor volume size (V) was determined as described in a previous study (Liu et al. 2021), where V = (L × W^2^)/2 (L denoting the long diameter and W denoting the short diameter). Nude mice were euthanized by cervical dislocation after 5 weeks. Tumor tissues were harvested for further histological examination. The animal experiments in this study were approved by the Animal Protection and Use Committee of Clinical Oncology School of Fujian Medical University and were conducted in compliance with the Chinese guidelines for ethical review of laboratory animal welfare.

### Hematoxylin–Eosin (HE) Staining

Subcutaneous tumor tissue sections embedded in paraffin (4 μm) were prepared and stained using a hematoxylin–eosin (H&E) staining kit (Solarbio, G1120, Beijing, China) based on the experimental protocol of the supplier. Then, the histological features of the subcutaneous tumor sections were observed under an inverted microscope (BX5, Olympus, Tokyo, Japan).

#### Immunohistochemical (IHC)

Subcutaneous tumor tissue Sects. (4 μm) were incubated with 1 mM Tris–EDTA buffer (Servicebio, G1203, Wuhan, China) at high temperature (125 °C, 103 kPa) for 18 min. The sections were incubated in 3% H_2_O_2_ for 10 min at ambient temperature to inactivate endogenous peroxidase. After washing, the sections were incubated in 10% goat serum (Solarbio, SL038, Beijing, China) for 30 min at room temperature, followed by the incubation with an anti-Ki-67 antibody (Abcam, ab15580, Cambridge, UK) overnight at 4 °C. Then, a sheep anti-mouse IgG secondary antibody (Mxb Biotechnologies, KIT-5020, Fuzhou, China) was used to label the sections for 1 h at room temperature. An IHC DAB signal development kit (Beyotime, P0202, Beijing, China) was used for target signal development for 3–5 min at room temperature. The excess dye was washed using tap water, and hematoxylin solution was applied for another 3-min staining. The stain was then washed off using 1% hydrochloric acid alcohol. After the dehydration with ethanol, the sections were emended in xylene, sealed with neutral resin, and photographed under a microscope (Leica Microsystems, DM1000, Wetzlar, Germany).

#### Statistical Analysis

SPSS 22.0 software (IBM, Armonk, NY, USA) was used for the statistical analysis. Results were expressed as the mean and standard deviation (mean ± SD). The correlation between DR6 expression levels of CRC tumor samples and the clinicopathological features of CRC patients was analyzed using the Chi-square (χ2) test. The sample size and statistical power were determined using Power/Sample Size Calculator (https://www.stat.ubc.ca/~rollin/stats/ssize/n2.html), with default α = 0.05, desired power = 0.80. The overall survival (OS) of CRC patients was assessed using the Kaplan–Meier method, and the difference between the survival curves was determined using the log-rank test. Two-tailed unpaired Student's *t*-test was used to compare the differences between the two conditions, while one-way ANOVA was adopted to compare the differences between multiple groups. *P* < 0.05 indicates a statistically significant difference.

## Results

### High Expression of DR6 is associated with a poor prognosis in CRC patients

**Fig. 1:**
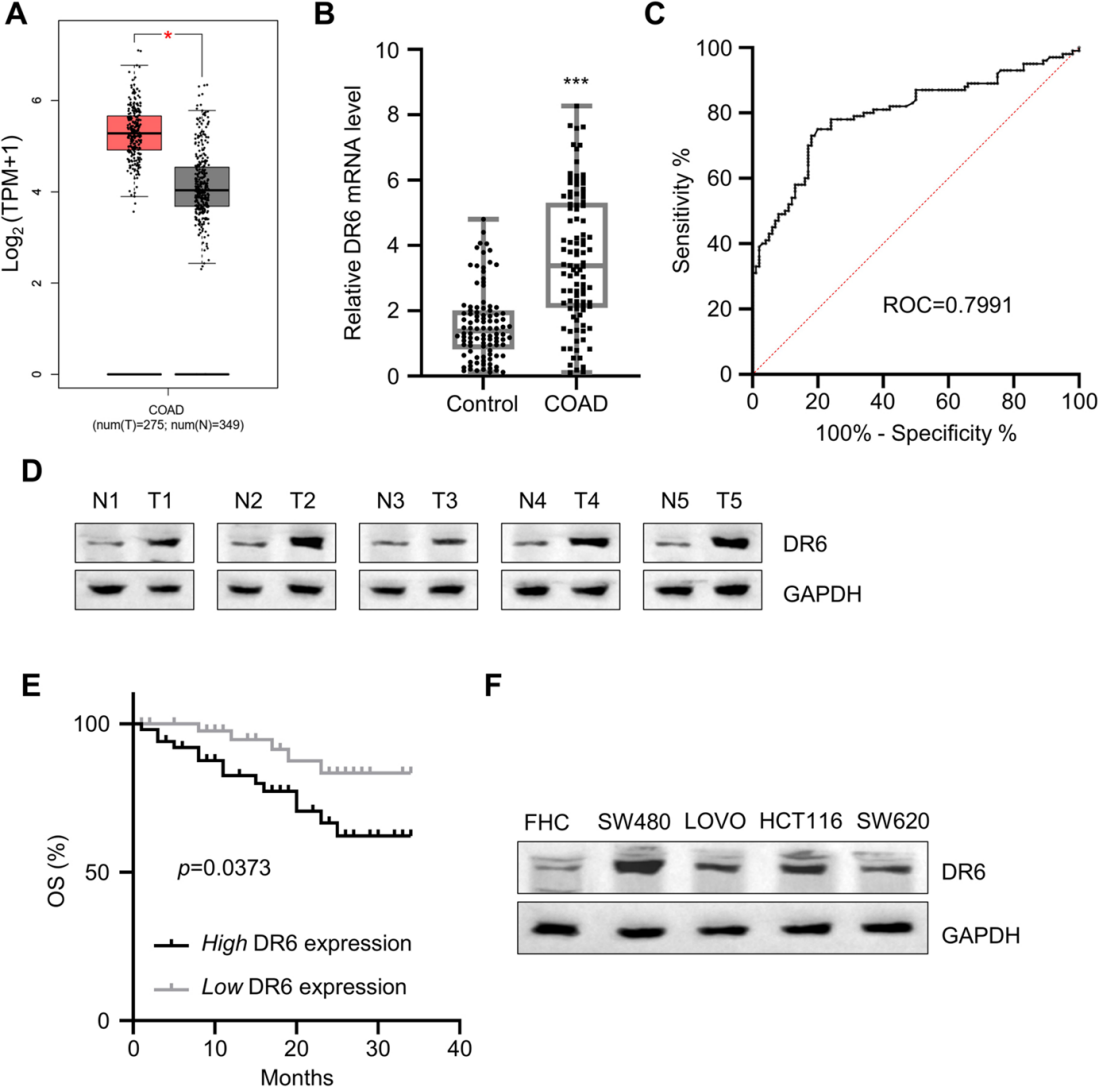
Elevated expression levels of DR6 in CRC tumor tissues and cells. **A** GEPIA online analysis of the TCGA-COAD dataset revealed the elevated expression of DR6 in CRC tumor samples. **B** qRT-PCR analysis of DR6 expression in 100 pairs of CRC tumor tissues and para-cancerous tissues. **C** ROC curve analysis of *DR6* mRNA levels as a diagnostic indicator of CRC. **D** Western blotting detection of DR6 protein levels in 5 pairs of CRC cancer tissues and para-neoplastic tissues. **E** The association between DR6 expression levels and the survival of CRC patients. CRC patients were divided into DR6 high-expression group (n = 50) and DR6 low-expression group (n = 50) according to the median DR6 expression level. Kaplan–Meier curves were used to analyze the overall survival (OS) of each group. **F** The relative protein levels of DR6 in CRC cells and FHC cells. ***P < 0.001, compared with the control group

Based on the median expression value of DR6 in CRC tumor tissues, we divided 100 CRC patients into two groups: DR6 high-expression group (n = 50) and DR6 low-expression group (n = 50). Chi-square test was conducted to analyze the association between DR6 expression levels and the clinicopathological data of CRC patients (Table 1). We found that the high-level expression of DR6 was positively associated with more advanced tumor stage, tumor invasion, and lymph node metastases. However, there was no correlation between DR6 levels and tumor size, age, or gender. Using the CRC cohort data from GEPIA database, we observed that there was a significantly higher level of DR6 expression in CRC tumor samples when compared to normal tissues (Fig. 1A). The upregulation of DR6 expression was further confirmed in the clinical samples of CRC tumors (Fig. 1B). According to the ROC curve analysis, the area under the curve (AUC) of DR6 was 0.8, indicating that DR6 expression level has a high prognostic value in distinguishing CRC tissues from normal tissues (Fig. 1C). Furthermore, Western blotting results also showed that DR6 protein levels were increased in CRC tumor tissues compared to para-neoplastic tissues (Fig. 1D). In addition, Kaplan–Meier curve analysis suggested that CRC patients with high DR6 expression suffered from a worse prognosis than the ones with low DR6 expression (Fig. 1E). There was also an upregulation of DR6 expression in CRC cancerous cell lines (HCT116, LOVO, SW480, and SW620) compared to the normal colonic epithelial cell line (FHC) (Fig. 1F). Together, these results suggest that DR6 overexpression is associated with a poor prognosis in CRC patients.

**Table 1:** Relationship between DR6 and clinicopathological factors of CRC patients

Characteristics	Number of cases	DR6 expression		*P* value
		Low (n = 50)	High (n = 50)	
Gender				0.4177
Male	58	27	31	
Female	42	23	19	
Age (years)				0.105
≤ 60	58	25	33	
> 60	42	25	17	
Tumor size (cm)				0.0163
≤ 5	52	32	20	
> 5	48	18	30	
Tumor invasion				0.0278
T1-T2	49	30	19	
T3-T4	51	20	31	
Lymphatic metastasis				0.044
Negative	56	33	23	
Positive	44	17	27	
Distant metastasis				0.0211
M0	86	47	39	
M1	14	3	11	
Clinical stage				0.0453
I-II	52	31	21	
III-IV	48	19	29

### Knockdown of DR6 Inhibits the Proliferation, Migration, Invasion, and Stemness of CRC Cells

**Fig. 2:**
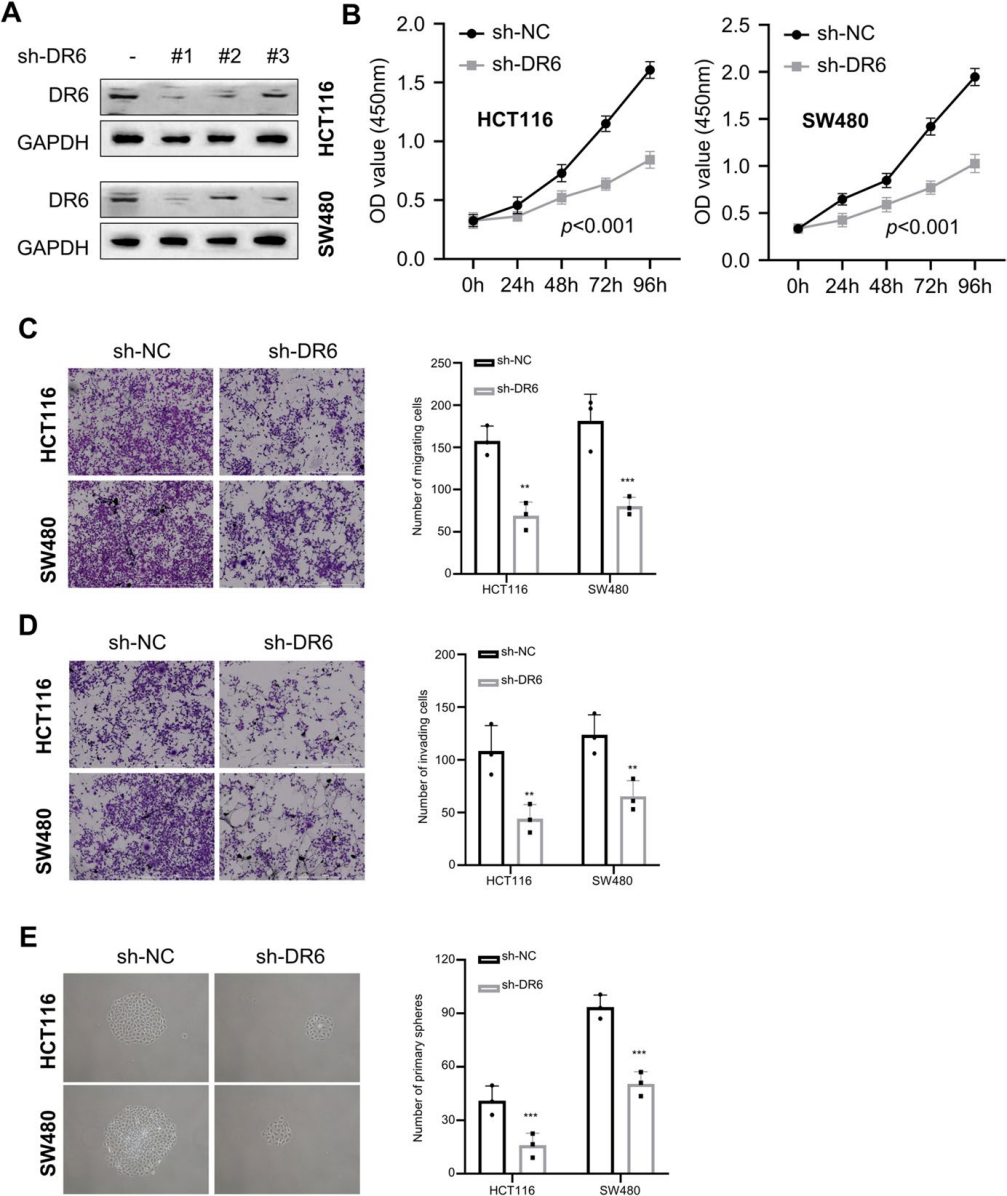
Knockdown of DR6 inhibits the proliferation, migration, invasion, and stemness of CRC cells. **A** Stable DR6 knockdown cell lines were established in HCT116 and SW480 cells using lentivirus. Cells were infected with lentivirus carrying sh-NC, sh-DR6-#1, -#2, and -#3. Western blot was conducted to detect the relative protein levels of DR6. sh-DR6 #1 showed the strongest knockdown efficiency and was selected as sh-DR6 for knockdown experiments. **B** CCK-8 proliferation assay in different groups of HCT116 and SW480 cells at 0 h, 24 h, 48 h, 72 h, and 96 h. **C** Transwell migration assay in HCT116 and SW480 cells with or without DR6 knockdown. **D** Transwell invasion assay in HCT116 and SW480 cells with or without DR6 knockdown. **E** Tumor spheroid-forming assay in HCT116 and SW480 cells with or without DR6 knockdown. **P < 0.01, ***P < 0.001, compared with the sh-NC group

To determine the functional significance of DR6 in CRC, we used lentivirus to stably knock down DR6 in HCT116 and SW480 cells, and the #1 DR6 targeting shRNA showed the strongest knockdown effect, which was used for the loss-of-function experiment (Fig. 2A). The CCK-8 proliferation assay showed that silencing DR6 suppressed cell proliferation in HCT116 and SW480 cells (Fig. 2B). The migratory and invasive abilities were also impaired upon DR6 silencing (Fig. 2C; 2D). Further, silencing DR6 reduced number of spheroids in the 3D culture system (Fig. 2E). These results indicate that DR6 knockdown impairs the proliferation, migration, invasion, and stemness of CRC cells.

### DR6 Overexpression Augments Cell Proliferation, Migration, Invasion, and Stemness of CRC Cells

**Fig. 3:**
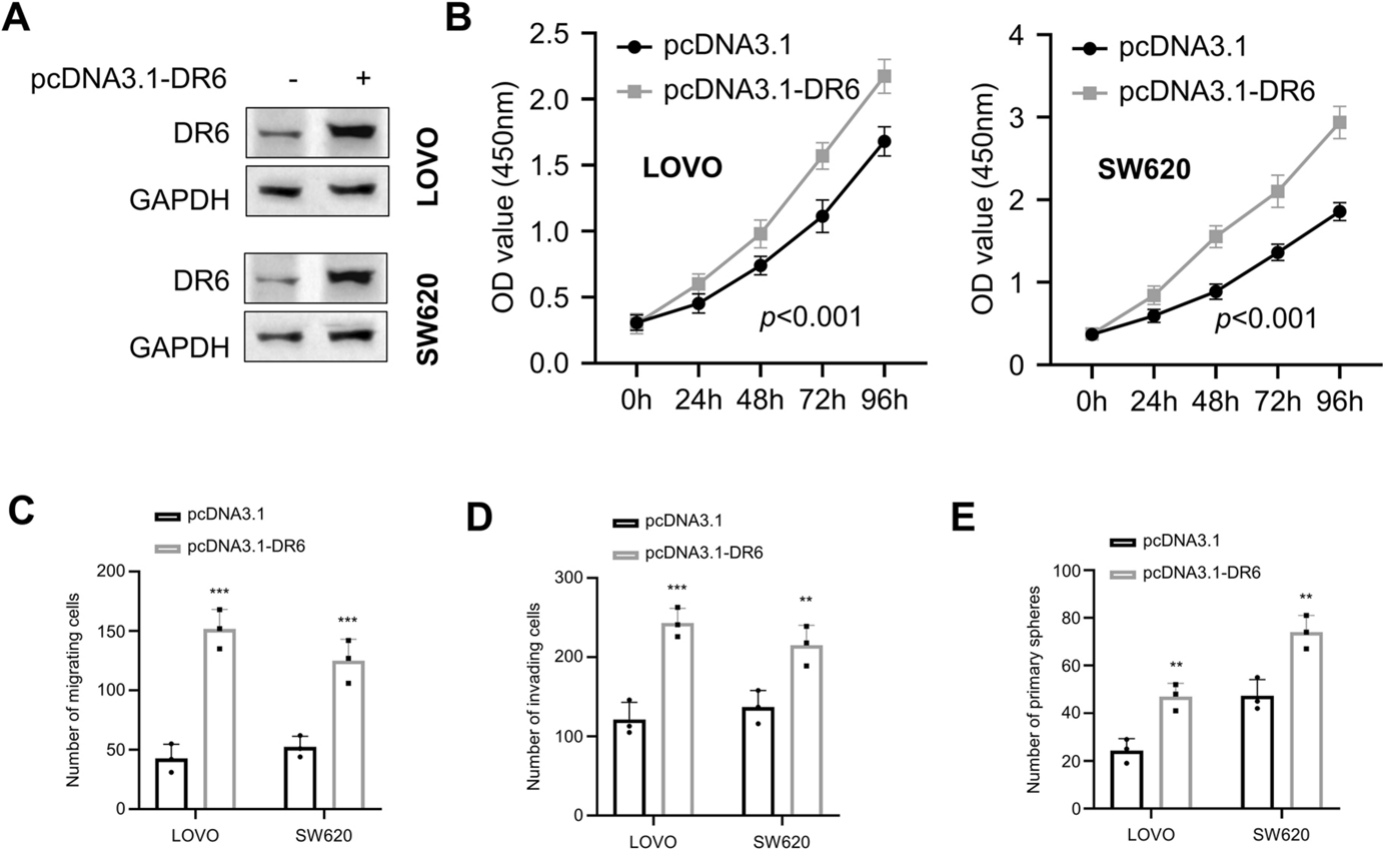
Overexpression of DR6 promotes the proliferation, migration, invasion, and stemness of CRC cells. **A** CRC cell lines were transfected with pcDNA3.1 empty vector or pcDNA3.1-RD6 plasmid, and the expression level of DR6 in LOVO and SW620 cells was detected by Western blotting. **B** CCK-8 proliferation assay in LOVO and SW620 cells with or without DR6 overexpression for 0 h, 24 h, 48 h, 72 h, and 96 h. **C** Transwell migration assay in LOVO and SW620 cells with or without DR6 overexpression. **D** Transwell invasion assay in LOVO and SW620 cells with or without DR6 overexpression. **E** Tumor spheroid-forming assay in LOVO and SW620 cells with or without DR6 overexpression. **P < 0.01, ***P < 0.001, compared with the pcDNA3.1 group

DR6 expression vector was used to overexpress DR6 in LOVO and SW620 cells, which exhibit comparatively diminished levels of DR6 expression (Fig. 1F; Fig. 3A). The CCK-8 proliferation assay demonstrated that cell growth ability was significantly enhanced upon DR6 overexpression (Fig. 3B). Transwell assays also showed the augmented cell migration and invasion upon DR6 overexpression (Fig. 3C; 3D). Further, DR6 overexpression also significantly enhanced the spheroid formation in the 3D culture system (Fig. 3E). These results suggest that DR6 overexpression could promote the malignant features of CRC cells.

### DR6 Promotes the Malignant Phenotypes of CRC Cells by Regulating the AKT/ NF-κB Pathway

**Fig. 4:**
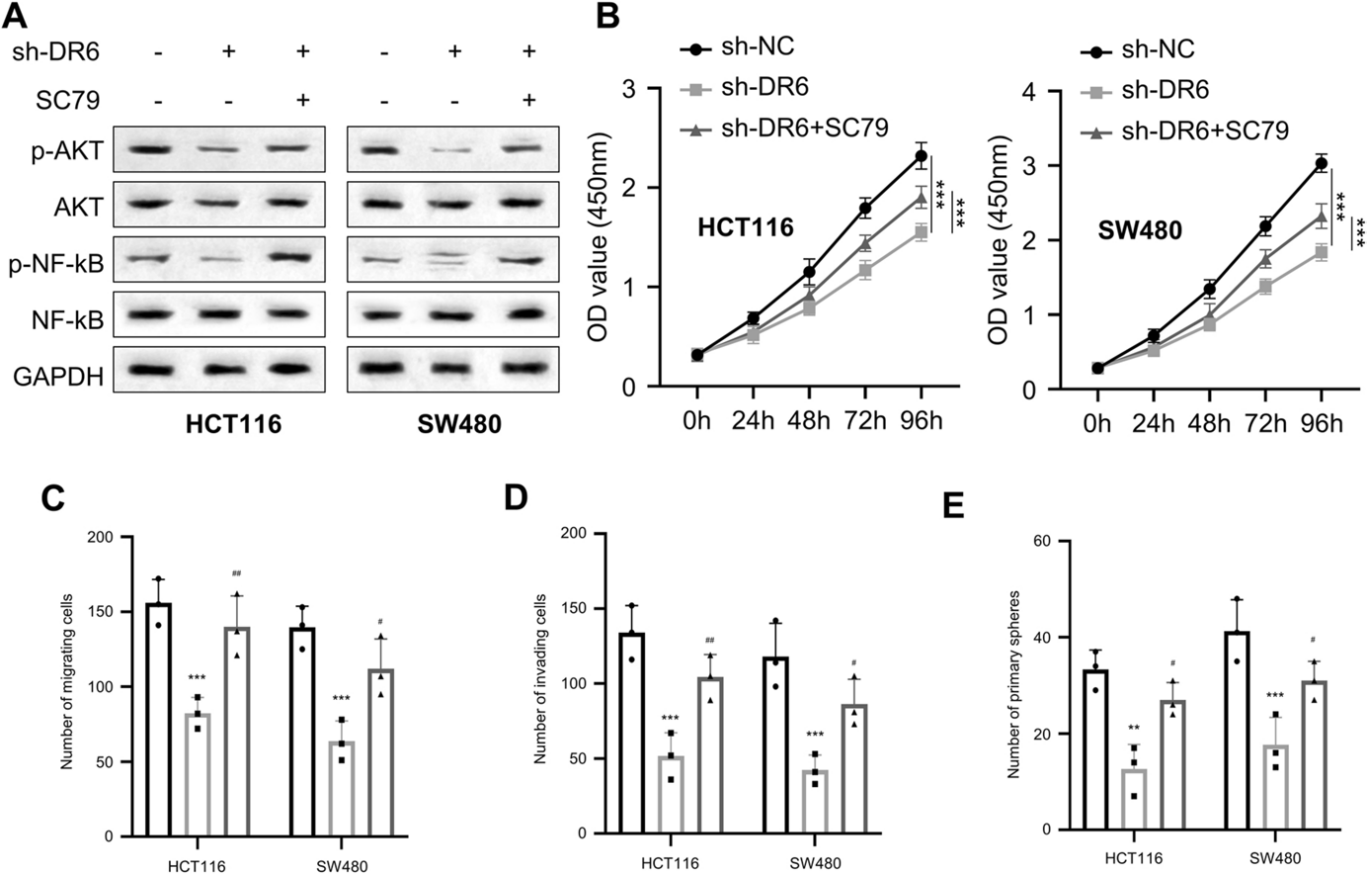
DR6 affects the malignant phenotype of CRC cells by regulating the AKT/NF-kB pathway. **A** Western blotting detection of p-AKT, AKT, p-NF-κB, and NF-κB in HCT116 and SW480 cells with or without DR6 silencing and AKT activator SC79 treatment. **B** CCK-8 proliferation assay in HCT116 and SW480 cells with or without DR6 knockdown and AKT activator SC79 treatment. **C** Transwell migration assay in HCT116 and SW480 cells with or without DR6 knockdown and AKT activator SC79 treatment. **D** Transwell invasion assay in HCT116 and SW480 cells with or without DR6 knockdown and AKT activator SC79 treatment. **E** Tumor spheroid-forming assay in HCT116 and SW480 cells with or without DR6 knockdown and AKT activator SC79 treatment. **P < 0.01, ***P < 0.001, compared with sh-NC group. #*P* < 0.05, ##*P* < 0.01, compared with the sh-DR6 + DMSO group

To examine whether AKT/ NF-κB pathway is involved in the functional role of DR6, we treated HCT116 and SW480 cells with the AKT activator SC79 after DR6 knockdown. DR6 silencing suppressed the phosphorylation of AKT and NF-κB proteins in HCT116 and SW480 cells, an effect which was rescued after the treatment of AKT activator SC79 (Fig. 4A). Furthermore, the application of AKT activator also promoted cell proliferation, migration, invasion, and spheroid formation in HCT116 and SW480 cells with DR6 knockdown (Fig. 4B-4E). These results indicate that silencing DR6 attenuates the malignancy of CRC cells through dampening the AKT/NF-κB signaling pathway.

### Knockdown of DR6 Inhibits the Tumorigenesis of CRC cells in vivo

**Fig. 5:**
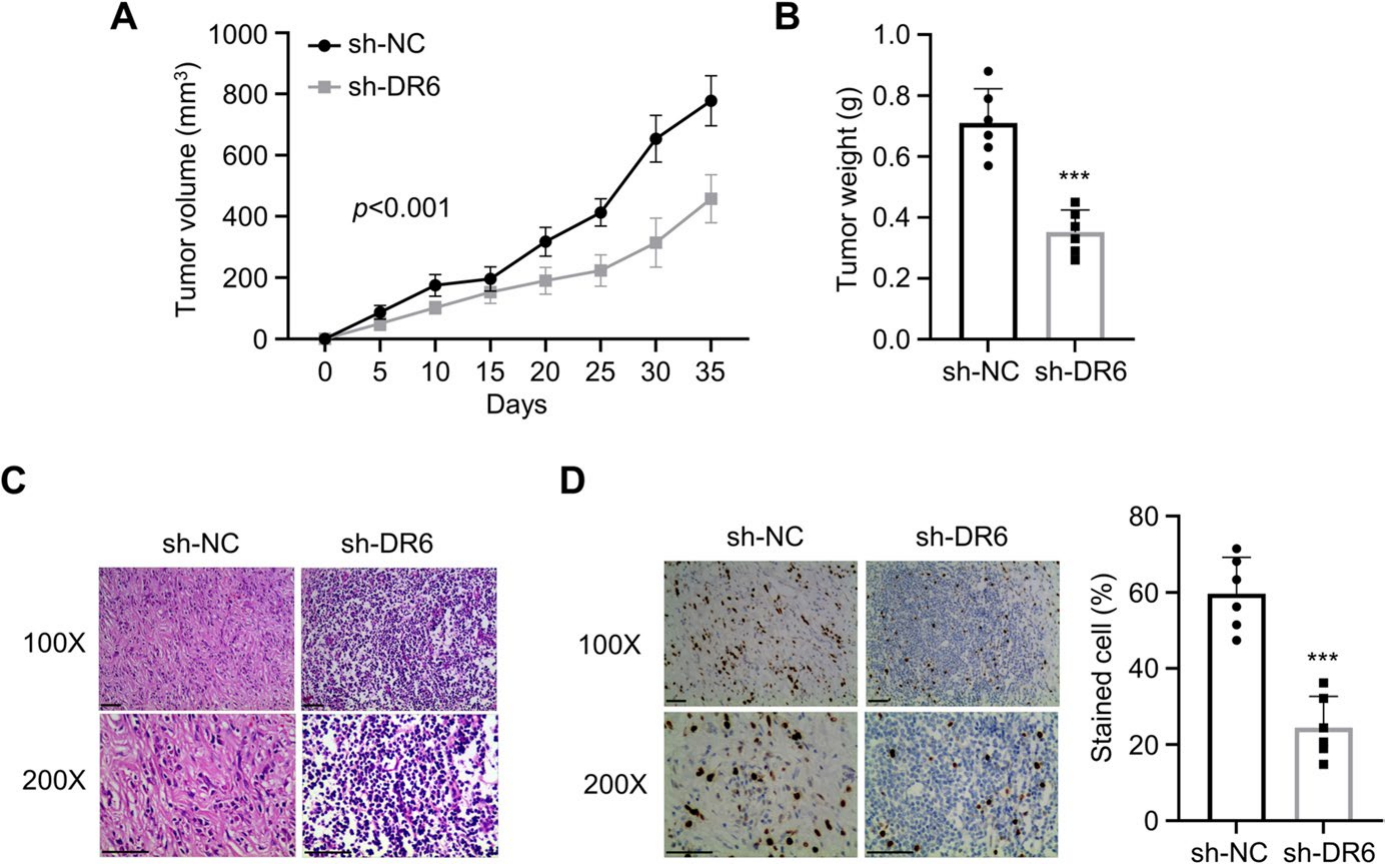
Knockdown of DR6 inhibits CRC cell growth in vivo*.* **A** Detection of subcutaneous tumor volume in sh-NC and sh-DR6 groups. **B** The summary of tumor weight in sh-NC and sh-DR6 groups. **C** HE staining in the tumor samples of sh-NC and sh-DR6 groups. **D** Immunohistochemical detection of Ki-67 protein in the tumor samples of sh-NC and sh-DR6 groups. ***P < 0.001

In addition, we injected HCT116 cells with stable expression of control shRNA (sh-NC) or DR6 targeting shRNA (sh-DR6) into nude mice to investigate the impact of DR6 knockdown on the tumorigenesis of CRC cells. DR6 knockdown caused a considerable reduction in tumor volume (Fig. 5A) and tumor weight (Fig. 5B). Furthermore, HE staining revealed a loosely packed tumor cells in the tumor samples from HCT116 cells with DR6 knockdown (Fig. 5C). Meanwhile, IHC staining showed that DR6 knockdown caused a decreased expression of cell proliferation marker Ki-67 in the tumor samples (Fig. 5D). These findings indicate that DR6 is required for the tumorigenesis of CRC cells in vivo.

## Discussion

DR6, also known as TNFRSF21, is a TNFRSF cell surface receptor involved in a variety of cellular processes, including cell growth, differentiation, and apoptosis (Hsu et al. 2022; Chen et al. 2019). DR6 and its related signaling pathways have been found to exert an oncogenic role in the malignant progression of cancers. Furthermore, abnormal DR6 expression has been suggested as a diagnostic or prognostic biomarker for some cancers, such as glioma, sarcoma, and ovarian cancer (Ren et al. 2022). However, the potential functional role of DR6 in CRC has not yet been investigated.

Based on online database analysis, we showed that DR6 expression was elevated in CRC tumor samples when compared to the normal counterparts, which was validated by qRT-PCR and Western blot analysis in our clinical samples. DR6 expression was also substantially upregulated in CRC cell lines than the normal human colonic epithelial cells. Further, increased DR6 expression was significantly associated with a poor prognosis in CRC patients. We also showed that DR6 knockdown suppressed the malignant features of CRC cells, while DR6 overexpression augmented the malignancy of CRC cells. Importantly, DR6 silencing also attenuated the proliferation and tumor formation of CRC cells in the animal model. Thus, our data suggest that DR6 functions as an oncogene to promote the malignant progression of CRC cells, which are consistent with the previous studies (Xu et al. 2022; Stegmann et al. 2022; Shi et al. 2018; Yang et al. 2012, 2016).

We further showed that silencing DR6 attenuated the malignancy of CRC cells by dampening the AKT/NF-κB signaling pathway, suggesting that DR6 is a positive regulator of AKT/NF-κB signaling in CRC cells. As the TNFRSF member, DR6 is a ligand-regulated transmembrane protein which impinges on cell survival through NF-κB signaling (Kasof et al. 2001). Aberrant activation of NF-κB signaling can promote cell proliferation, migration, and invasion of CRC cells (Zhu et al. 2020). This is consistent with our finding that DR6 may promote the malignancy of CRC cells through activating NF-κB signaling. DR6 is required for the angiogenesis of mouse B16 tumor via the NF-κB, P38 MAPK, and STAT3 pathways (Yang et al. 2016). It has been well documented that NF-κB signaling interacts with different cellular signaling pathways to facilitate cancer progression, the development of drug resistance, and metastasis (Labouba et al. 2015; Xia et al. 2014). For example, the activation of NF-κB and STAT3 pathways promotes the expression of anti-apoptotic, pro-proliferative, and immune response genes, and some of these response genes require the transcriptional cooperation between the two signaling pathways (Grivennikov and Karin 2010). The interaction between STAT3 and NF-κB also plays a crucial role in the dialogue between cancer cells and the tumor microenvironment, which facilitates the immune cell infiltration (Grivennikov and Karin 2010). Additionally, the crosstalk between NF-κB and other pathways, including insulin-like growth factor pathway, reactive oxygen species signaling, and Notch signaling pathway, has also been wildly reported to impinge on the malignant progression of cancers (Maniati et al. 2011; Morgan and Liu 2011; Li et al. 2015). Similarity, AKT is a downstream effector of PI3K signaling, and the PI3K/AKT signaling is frequently activated in different cancers to facilitate the aggressive progression and confer drug resistance (Rascio et al. 2021). The coordinated activation of PI3K/AKT and NF-κB Signaling can drive the malignant progression of multiple tumors including CRC (Ahmad et al. 2013), and these pathways are important targets for anti-cancer therapy. The crosstalk between PI3K/AKT and MAPK signaling pathways is also implicated in the pathogenesis of CRC (Stefani et al. 2021). Therefore, the activation of NF-κB and AKT signaling by DR6 may also foster the malignant progression of CRC through the crosstalk with other signaling cascades.

Different molecular players have been identified to regulate the progression of CRC cells due to the application of next generation sequencing technologies. For instance, non-coding RNAs such as lncRNAs, circRNAs, and miRNAs have been reported to be dysregulated and implicated in the progression of CRC (Bin et al. xxxx; Su et al. xxxx; Liu et al. xxxx; Shen et al. xxxx; Zhang et al. xxxx; Chen et al. xxxx). The deregulation of these non-coding RNAs targets different downstream mRNAs and signaling pathways to modulate CRC progression. Besides, there is increasing evidence that a combination of different biomarkers is of great potential to serve as diagnostic and prognostic tools in CRC (Jamai, et al. xxxx; Saucedo-Sariñana et al. xxxx). Whether DR6 can be employed as a reliable prognostic and metastatic biomarker in CRC management warrants future validation in a prospective study in a large cohort of patients. Further, different diet and drug regimens (such as curcumin, soy isoflavones, and mFOLFOX6 regimen) have been reported to have potential in improving the prognosis of CRC patients (Yu et al. xxxx; Sun et al. xxxx). Whether these regimens also target DR6 and its downstream signaling pathways remain to be clarified.

In conclusion, we demonstrated that DR6 stimulates AKT/NF-κB pathway to promote the malignant features of CRC cells, including the enhancement of cell proliferation, migration, invasion, and cell stemness. These data suggest that DR6 might be employed as a diagnostic and predictive biomarker for CRC progression, as well as a molecular target for developing novel therapy for CRC. In future investigations, the molecular mechanisms of DR6 in dictating the EMT-related signaling and metastatic potential of CRC cells need to be further clarified.

## Data Availability

All data can be obtained by request.
